# High-Yield Preparation and Electrochemical Properties of Few-Layer MoS_2_ Nanosheets by Exfoliating Natural Molybdenite Powders Directly via a Coupled Ultrasonication-Milling Process

**DOI:** 10.1186/s11671-016-1622-3

**Published:** 2016-09-19

**Authors:** Huina Dong, Deliang Chen, Kai Wang, Rui Zhang

**Affiliations:** 1 School of Materials Science and Engineering, Zhengzhou University, Zhengzhou, 450001 People’s Republic of China; 2 School of Chemical Engineering and Energy Technology, Dongguan University of Technology, Dongguan, 523808 People’s Republic of China; 3 Laboratory of Aeronautical Composites, Zhengzhou Institute of Aeronautical Industry Management, Zhengzhou, 450046 People’s Republic of China

**Keywords:** Coupled ultrasonication-milling process, Multi-forces synergistic exfoliation, Natural molybdenite powders, Few-layer MoS_2_ nanosheets, Liquid-phase exfoliation, Electrochemical performance, Layered materials

## Abstract

Cost-effective and scalable preparation of two-dimensional (2D) molybdenum disulfide (MoS_2_) has been the bottleneck that limits their applications. This paper reports a novel coupled ultrasonication-milling (CUM) process to exfoliate natural molybdenite powders to achieve few-layer MoS_2_ (FL-MoS_2_) nanosheets in the solvent of *N*-methyl-2-pyrrolidone (NMP) with polyvinylpyrrolidone (PVP) molecules. The synergistic effect of ultrasonication and sand milling highly enhanced the exfoliation efficiency, and the precursor of natural molybdenite powders minimizes the synthetic cost of FL-MoS_2_ nanosheets. The exfoliation of natural molybdenite powders was conducted in a home-made CUM system, mainly consisting of an ultrasonic cell disruptor and a ceramic sand mill. The samples were characterized by X-ray diffraction, UV-vis spectra, Raman spectra, FT-IR, SEM, TEM, AFM, and N_2_ adsorption-desorption. The factors that influence the exfoliation in the CUM process, including the initial concentration of natural molybdenite powders (*C*_in_, 15–55 g L^−1^), ultrasonic power (*P*_u_, 200–350 W), rotation speed of sand mill (*ω*_s_, 1500–2250 r.p.m), exfoliation time (*t*_ex_, 0.5–6 h), and the molar ratio of PVP unit to MoS_2_ (*R*_pm_, 0–1), were systematically investigated. Under the optimal CUM conditions (i.e., *C*_in_ = 45 g L^−1^, *P*_u_ = 280 W, *ω*_s_ = 2250 r.p.m and *R*_pm_ = 0.5), the yield at *t*_ex_ = 6 h reaches 21.6 %, and the corresponding exfoliation rate is as high as 1.42 g L^−1^ h^−1^. The exfoliation efficiency of the CUM mode is much higher than that of either the ultrasonication (U) mode or the milling (M) mode. The synergistic mechanism and influencing rules of the CUM process in exfoliating natural molybdenite powders were elaborated. The as-obtained FL-MoS_2_ nanosheets have a high specific surface area of 924 m^2^ g^−1^ and show highly enhanced electrocatalytic performance in hydrogen evolution reaction and good electrochemical sensing property in detecting ascorbic acid. The CUM process developed has paved a low-cost, green, and highly efficient way towards FL-MoS_2_ nanosheets from natural molybdenite powders.

## Background

Since the discovery of graphene, two-dimensional (2D) nanomaterials have attracted highly increasing attention owing to their unique few-layer microstructures and resulting unprecedented functional properties [1–4]. Among them, the most typical 2D sample should be transition-metal dichalcogenides (TMDs), composed of covalently bonded transition-metal atoms (M) and chalcogen atoms (X = S, Se, Te) in a possible stoichiometric form of MX_2_ [5], such as MoS_2_ and WS_2_, of which each single-layer consists of two S atom layers and a layer of metal atoms sandwiched between the two S layers [6]. Owing to the suitable intrinsic indirect band gap (1.3 eV), MoS_2_ has attracted great attention and been thought as alternative promising next-generation 2D materials. When the layered MoS_2_ bulks are exfoliated into single-layered 2D nanosheets, their band gap increases from 1.3 to 1.9 eV [7, 8]. The interesting nanostructures and band gaps make MoS_2_ a useful active material in various fields, including energy storage and solar cells [9–14], catalysis [15–19], sensing [19–21], and electronic devices [22–24]. As a major member of the TMD family, MoS_2_ has been known as a highly efficient catalyst for the hydro-desulfurization, reduction reaction of methyl orange by MoS_2_/montmorillonite [25] and hydrogen evolution reactions (HER) [26–28]. Experimental and theoretical studies have verified that the catalytic activity of MoS_2_ is associated with metallic edges, whereas its semiconducting basal plane is catalytically inert [29]. Few-layer MoS_2_ (FL-MoS_2_) nanosheets can maximize the fraction of exposed edge sites and thus show highly enhanced catalytic performance [30]. FL-MoS_2_ nanosheets can also be used as electrochemical biosensors for the detection of glucose [31], acetaminophen (AC) [32], double-stranded DNA [33], ascorbic acid [34], and H_2_O_2_ [35] which show a high response due to the advantage of abundant exposed edges.

Scalable and cost-effective synthesis of high-quality FL-MoS_2_ nanosheets is one of the preconditions for their commercial applications. Up to now, the available methods for the preparation of monolayer or FL-MoS_2_ nanosheets can be categorized into two types: “bottom-up” growth and “top-down” exfoliation. For the “bottom-up” growth, solvothermal methods [36–38], chemical vapor deposition [39, 40], and thermal decomposition [41, 42] have been developed to synthesize MoS_2_ nanocrystals with various shapes and sizes using molybdenum-containing chemicals as the Mo source. Unfortunately, the molybdenum-containing chemicals are expensive, and the morphology and size of MoS_2_ nanocrystals in the “bottom-up” growth are not easy to control. The “top-down” exfoliation, starting from three-dimensional (3D) layered bulks, is commonly used to prepare 2D nanomaterials. FL-MoS_2_ nanosheets have been synthesized by the “top-down” exfoliation, including micromechanical exfoliation [43, 44], chemical exfoliation through intercalation [45–48], electrochemical exfoliation [49, 50], liquid-phase exfoliation (LPE) [51–58], grinding-assisted liquid exfoliation [59–61], and supercritical fluid exfoliation [62].

Micromechanical exfoliation, firstly developed to prepare graphene by exfoliating HOPG [63], can achieve isolated individual crystal planes (monolayers) with high crystal quality and macroscopic continuity, but its yield is too low to be of any practical applications. Chemical exfoliation usually necessitates intercalating various small species (i.e., organic molecules, alkali metals and transition-metal halides) into the interlayer spaces to achieve the exfoliation. In most cases, chemical exfoliation is time-consuming, sensitive to environmental conditions, and difficult to control the intercalated degree [49]. Electrochemical exfoliation, through a controllable lithiation process, has been developed to exfoliate the layered solids (i.e., MoS_2_, WS_2_, TiS_2_, TaS_2_, ZrS_2_, and graphite) as the cathode in an electrochemical setup, and the lithium-intercalation in the layered materials can be monitored and finely controlled during the discharge process [49], but high-cost and low yield limit its wide applications. Liquid-phase exfoliation (LPE) is one of the most common methods used in the exfoliation of layered solids recently [51]. The LPE process involves the following steps: (1) immersion of layered materials in a suitable solvent (i.e., NMP, DMF, THF, NVP, CAN, and DMSO), (2) exfoliation of layered materials, and (3) stabilization of exfoliated 2D materials [51]. Sufficient liquid immersion is one of the most effective and straightforward means to reduce the strength of the van der Waals attractions, where the potential energy between adjacent layers is mainly contributed by the dispersive London interactions. Although LPE can be scaled up, the solvents used are usual toxic and not environmentally friendly. Supercritical fluid exfoliation is the newly developed method to prepare few-layer nanosheets via the shearing force generated by the gas molecules in the supercritical condition [64]. To further improve the exfoliated efficiency, scientists have coupled various methods, i.e., the two-solvent grinding-assisted LPE process [65] and ionic liquid assisted grinding [35, 66], to exfoliate layered solids to make few-layer nanosheets. However, scalable, efficient, cost-effective, and high-yield synthesis of FL-MoS_2_ nanosheets is still challengeable.

**Fig. 1:**
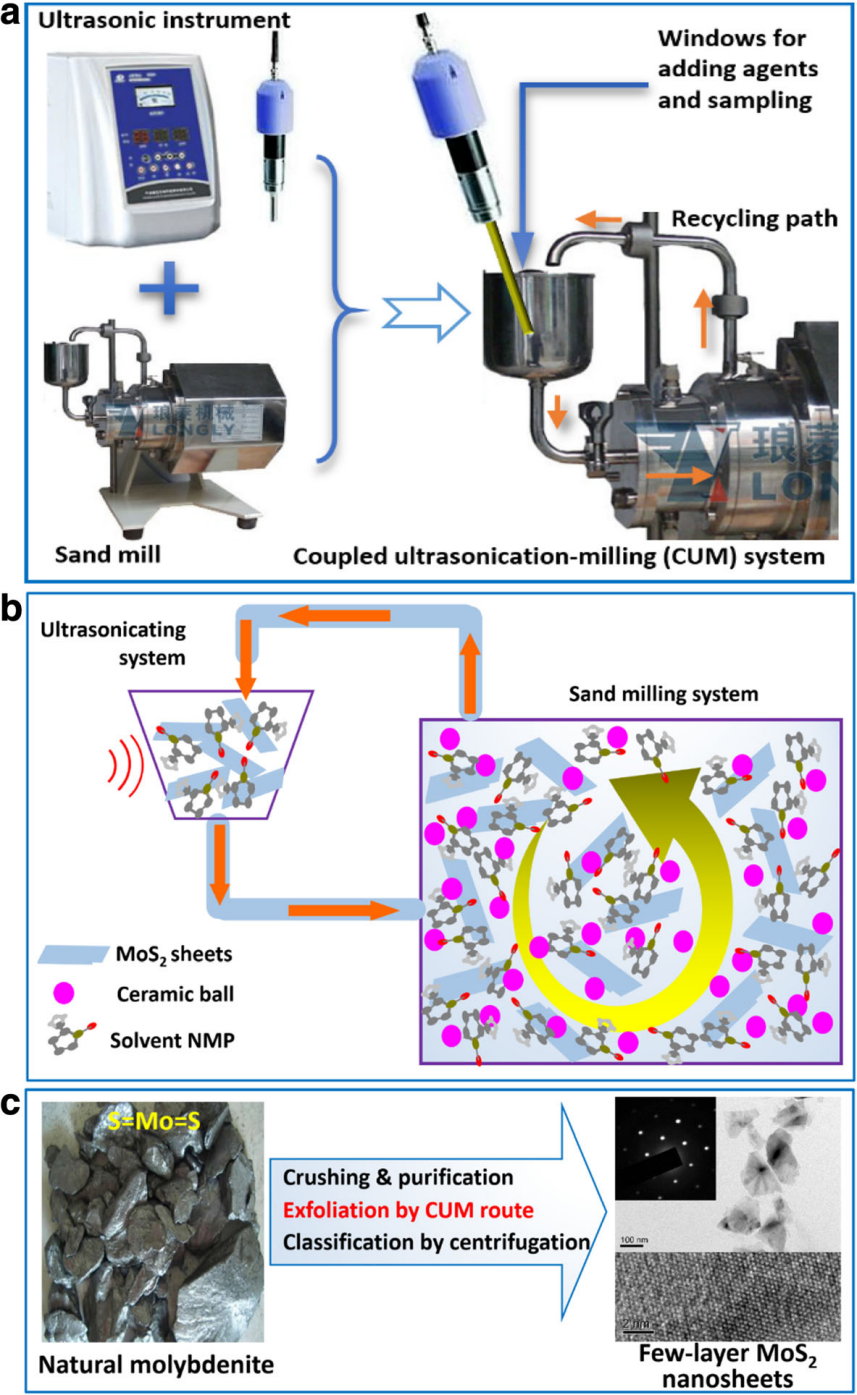
Schematic diagram of the coupled ultrasonication-milling (CUM) strategy for exfoliating natural molybdenite powders to make few-layer MoS_2_ (FL–MoS_2_) nanosheets: **a** Components of the CUM system, **b** basic operational principle of the CUM system, and **c** exfoliation of natural molybdenite towards FL-MoS_2_ nanosheets by the CUM route

Here, we develop a novel coupled ultrasonication-milling (CUM) process, as shown in Fig. 1, to exfoliate natural molybdenite powders to prepare FL-MoS_2_ nanosheets in a high-efficient manner via the synergistic effect of ultrasonication and sand milling. The precursor of natural molybdenite powders is directly from the molybdenite ore after a purification and superfine treatment. Molybdenite ore is abundant in China, and using natural molybdenite as the starting material can achieve a low-cost and low-carbon approach to make FL-MoS_2_ nanosheets. For the ultrasonic (U) exfoliation, the key point is the choice of suitable exfoliation solvent, and it is favorable to exfoliation when the surface tension of the solvent matches well with the surface energy of the layered bulk materials. The surface energy of MoS_2_ reported is ~75 mJ m^−2^, and the *N*-methyl-2-pyrrolidone (NMP) with a surface tension of 41 mN m^−1^ is found to be a good solvent for the exfoliation and dispersion of MoS_2_ nanomaterials [67]. NMP is therefore chosen as the exfoliation solvent in the exfoliation of natural molybdenite powders via the present CUM process. Because of the steric effect of polyvinylpyrrolidone (PVP) molecules, the exfoliating yield of FL-MoS_2_ via the CUM process with the aid of PVP under the optimal conditions reaches 21.6 %, much higher than those of the reported methods. The as-obtained FL-MoS_2_ nanosheets by the CUM process from natural molybdenite powders show highly electrochemical performance for hydrogen evolution reaction and sensing applications. The newly developed CUM process combines the advantage of both liquid-phase exfoliation (LPE) and mechanical exfoliation. The synergistic effect of the shear force generated by the high-speed sand mill and the cavitation by the liquid-phase ultrasonic process can highly accelerate the exfoliation of molybdenite powders. The key points of this work are to explore the influencing factors and possible synergistic effect during the CUM process.

## Methods

### Materials and Setup

Natural molybdenite powders (particle size 3–5 μm; MoS_2_ content ≥99 %; Luoyang Exploiter Molybdenum Co. LTD, China), *N*-methyl-2-pyrrolidone (NMP, ≥ 99 %; DAMAO Tianjin), and polyvinyl pyrrolidone (PVP, Molecular weight 1000–1,300,000, AR, Kermel) were used as received. The CUM system was home-made, mainly consists of an ultrasonic processor (Nanjing Atpio Instruments Manufacturer, China) and a sand mill (NT-0.3 L, Longly, China), schematically shown in Fig. 1.

### Exfoliation of Natural Molybdenite Powders by CUM Process

The exfoliation of natural molybdenite powders towards FL-MoS_2_ nanosheets was conducted in the home-made CUM system (Fig. 1) using NMP as the solvent containing PVP as the exfoliation agent at ~25 °C. The exfoliation of natural molybdenite powders by the CUM process was conducted under the similar conditions. Typically, natural molybdenite powders (~11.25 g) and PVP (~3.9 g) were dispersed in 250 mL of NMP, forming a mixture with an initial molybdenite concentration of 45 g L^−1^ and a PVP concentration of 15.6 g L^−1^. The above mixture was ultrasonically treated for ~20 min and then was poured into the sand mill. The synergistic exfoliation under milling and cavitation was kept for 4 h using the CUM process (Fig. 1). The diameter of the horn in the ultrasonication system was 10 mm, and the ultrasonication power was 280 W. The ultrasonication acted intermittently, i.e., 3 s on and 1 s off, avoiding damage to the ultrasonic processor because of heating. The rotation speed of the sand mill was kept at 2250 r.p.m. The as-obtained FL-MoS_2_ suspension was classified by centrifugation at various speeds to achieve FL-MoS_2_ dispersions. Some of the FL-MoS_2_ dispersions were centrifuged, washed carefully, and then freeze-dried to achieve FL-MoS_2_ nanosheets.

The initial concentration of natural molybdenite powders (*C*_in_,15–55 g L^−1^), ultrasonic power (*P*_u_, 200–350 W), rotation speed of sand mill (*ω*_s_, 1500–2250 r.p.m), exfoliation time (*t*_ex_, 0.5–6 h), and the molar ratio of PVP unit to MoS_2_ (*R*_pm_, 0–1) were chosen as the major influencing factors that were investigated systematically during the exfoliation. The exfoliation rate (*R*_ex_, g L^−1^ h^−1^) of FL-MoS_2_ nanosheets was measured by the FL-MoS_2_ concentration (*C*_ti_) in the upper dispersion after centrifuging the exfoliated suspension with a standing (more than ~24 h) at 1500 r.p.m for 45 min: *R*_ex_ = (*C*_t1_ − *C*_t2_)/Δ*t* = (*C*_t1_ − *C*_t2_)/(*t*_1_ − *t*_2_), where *C*_t1_ and *C*_t2_ were the FL-MoS_2_ concentrations of the upper dispersions obtained at exfoliation time *t*_1_ and *t*_2_, respectively. The exfoliation yield was defined as *α* = *C*_1_/*C*_0_ × 100 %, where *C*_1_ was the FL-MoS_2_ concentration of the upper dispersion at the ending, and *C*_0_ was the initial concentration of natural molybdenite powders. The FL-MoS_2_ concentration in the upper suspension was determined via the absorption coefficient (*ε*) at 670 nm according to the Beer-Lambert law (*A*/*l* = *ε C*) with a typically known concentration value (*C*’, g L^−1^). The concentration *C* was obtained by filtrating a certain amount (*V*, mL) of the target suspension, followed by washing, drying, and weighing the as-obtained solid species (*m*, mg): *C*’ = *m*/*V* [44]. The typical sample was made under the following parameters: *R*_pm_ = 1/2, *C*_in_ = 45 g L^−1^, *P*_u_ = 280 W, *ω*_s_ = 2250 r.p.m, and *t*_ex_ = 4 h. The FL-MoS_2_ dispersions and FL-MoS_2_ nanosheets were also prepared by the CUM process under various parameters (*C*_in_, *P*_u_, *ω*_s_, *t*_ex_, *R*_pm_) to investigate influencing factors.

In order to measure the exfoliation rate, a series of suspensions were sampled out for every 30 min and allowed to staying for ~24 h before centrifugation at 1500 r.p.m for 45 min. The absorption coefficients of the upper dispersions (2 mL) obtained by centrifugation were measured by UV-vis-IR absorption spectra. The concentrations of FL-MoS_2_ nanosheets in the upper dispersions were then calculated using the Beer-Lambert law.

For the purposes of comparison, the natural molybdenite powders were also exfoliated only using the ultrasonication or milling process. For the ultrasonication exfoliation (i.e., the U mode), 200 mL of NMP containing natural molybdenite powders (45 g L^−1^) and PVP (15.6 g L^−1^) was put in a wide-mouth bottle (250 mL) and ultrasonically treated for 4 h (280 W, 3 s on and 1 s off) using a φ10-mm horn. For the milling exfoliation using a sand mill (i.e., the M mode), the NMP mixture containing natural molybdenite powders (45 g L^−1^) and PVP (15.6 g L^−1^) was milled for 4 h using a rotation speed of 2250 r.p.m. Some of the MoS_2_ dispersions obtained by centrifugation at 1500 r.p.m for 45 min after standing for about 24 h were centrifuged once again at various speeds (e.g., 8000 r.p.m, 11,000 r.p.m) to achieve FL-MoS_2_ dispersions for further characterization and applications.

### Characterization of FL-MoS_2_

The UV-vis spectra of the FL-MoS_2_ suspensions were recorded on a UV-1800PC spectrometer (Shanghai Mapada), and a quartz cell with a path length of 1.0 cm was used as the sample pool. The SEM images were obtained using a JEOL JSM-7500F scanning electron microscope, with an Au film coating (20 mA for 50 s) before SEM observation. The TEM images were measured using a field-emission transmission electron microscope (JEOL, JEM-2100) with an accelerating voltage of 200 kV. The Raman spectra were obtained using a Raman spectrometer (LabRAM HR Evolution, HORIBA JobinYvon, France) at 525 nm, and the powder samples were prepared on a SiO_2_/Si substrate. The X-ray diffraction (XRD) patterns were recorded on an XD-3 X-ray diffractometer (Beijing Purkinje General Instrument Co., Ltd., China) with a Cu Kα irradiation (*λ* = 0.15406 nm). The Fourier transformed infrared (FT-IR) spectra were recorded on a Bruker-Equinox 55 spectrometer in a transmittance mode in a wavenumber range of 4000 to 400 cm^−1^. The atomic force microscopy (AFM) measurement was performed on a Bruker NanoScope V instrument in a tapping mode. The N_2_ adsorption-desorption isotherms were obtained on a Surface Area and Porosity Analyzer (ASAP 2460, Micromeritics).

### Test of Electrochemical Property

The electrochemical property of the FL-MoS_2_ nanosheets was characterized as electrocatalysts for hydrogen evolution reaction (HER) and as an active material for electrochemical sensors, respectively. For electrocatalytic applications, the upper FL-MoS_2_ dispersion obtained by centrifugation at 8000 r.p.m for 45 min was centrifuged once again at 10000 r.p.m for 45 min, and the as-obtained solid FL-MoS_2_ sample was used for HER test. To prepare electrodes, 4 mg of the FL-MoS_2_ nanosheets was dispersed in a mixture of Nafion (50 μL) and *N*, *N*-dimethylformamide (DMF, 950 μL) by sonication for more than 30 min to form a homogeneous ink. Before modification, the glassy carbon electrode (GCE) with a diameter of 3 mm was polished to a mirror finish using polish paper and alumina pastes of 50–70 μm and 30–50 nm, respectively, and then cleaned in an ultrasonic cleaner with acetone, alcohol, and water. Finally, 4 μL of the FL-MoS_2_ ink was carefully loaded onto the surface of the above GCE electrode to prepare electrodes modified with FL-MoS_2_ by allowing the liquids to evaporate under infrared light.

All electrochemical experiments were performed with a three-electrode system in which the Pt-wire electrode, saturated calomel electrode (SCE), and FL-MoS_2_-modified GCE (0.07 cm^2^) electrode were used as the counter, reference, and working electrodes, respectively. All measurements were carried out at room temperature. The HER activity was evaluated by linear sweep voltammetry conducted with a electrochemical working station (model CS310, Wuhan Correst Instruments Co., Ltd) with a scan rate of 2 mV s^−1^. The potential values with the reference of SCE electrode were calculated to be reverse hydrogen electrode (RHE): *E*_(RHE)_ = *E*_(SCE)_ + 0.241 V, in 0.5 M H_2_SO_4_. Cyclic voltammograms (CVs) were recorded in a potential range of −1.0–1.0 V to characterize the electrochemical sensing response to ascorbic acid (VC) at a potential scan rate of 20–150 mV s^−1^ in a solution consisting of 0.1 M KCl and ascorbic acid with different concentrations.

## Results and Discussion

### Exfoliation of Natural Molybdenite via the CUM Process

The exfoliation of natural molybdenite powders was conducted in a home-made coupled ultrasonication-milling (CUM) system. As Fig. 1a shows, this system consists of an ultrasonic instrument and a sand mill that are commercially available. Possible working mechanism in this CUM system can be described as Fig. 1b. Its unique feature is that the sand milling and ultrasonication can act at the same time. The major difference of between conventional wet ball milling and sand milling is their rotation speeds. The rotation speed of sand milling in this exfoliation is more than 2000 r.p.m, much higher than that of the conventional wet ball milling. Also, the special recycling in the sand milling can improve the exfoliation efficiency. With the CUM system, natural molybdenite powders can be exfoliated to few-layer MoS_2_ nanosheets via classification by centrifuging, as shown as Fig. 1c.

**Fig. 2:**
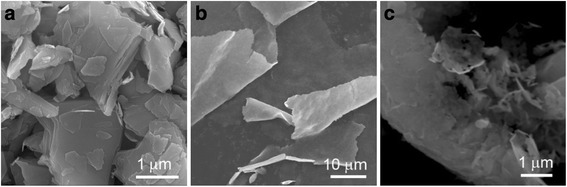
SEM images of **a** the pristine natural molybdenite powders and **b**, **c** the FL-MoS_2_ nanosheets obtained by centrifugation at 5000–8000 r.p.m (*R*_pm_ = 1/2, *C*_in_ = 45 g L^−1^, *P*_u_ = 280 W, *ω*_s_ = 2250 r.p.m, *t*_ex_ = 4 h)

Natural molybdenite powders were exfoliated to FL-MoS_2_ via the CUM process using the synergistic effect of ultrasonic cavitation and shearing force (Fig. 1a). Figure 2 shows the SEM images of the pristine molybdenite powders and the FL-MoS_2_ nanosheets (*R*_pm_ = 1/2, *C*_in_ = 45 g L^−1^, *P*_u_ = 280 W, *ω*_s_ = 2250 r.p.m, *t*_ex_ = 4 h). As Fig. 2a shows, the natural molybdenite consists of micro-scale powders with an irregular morphology and their particle sizes range 1–3 μm. Figure 2b shows the SEM image of the typical FL-MoS_2_ nanosheets, which were assembled to be thin films during the centrifugation by overlapping one another. The enlarged SEM image shown in Fig. 2c indicates that the FL-MoS_2_ nanosheets in the thin films are flexible nanosheets with a lateral length of several hundred nanometers.

**Fig. 3:**
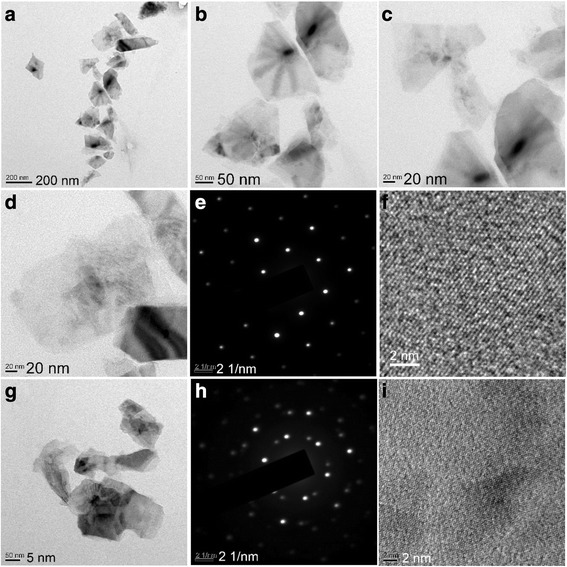
Typical TEM observations of the FL-MoS_2_ nanosheets derived from natural molybdenite (*R*_pm_ = 1/2, *C*_in_ = 45 g L^−1^, *P*_u_ = 280 W, *ω*_s_ = 2250 r.p.m, *t*_ex_ = 4 h): **a**–**d**, **g** low-magnification TEM images, **e**, **h** SAED patterns, and **f**, **i** HRTEM images

Figure 3 shows the typical TEM observations of the FL-MoS_2_ nanosheets derived from natural molybdenite after centrifugation at 8000–10,000 r.p.m via the CUM process (*R*_pm_ = 1/2, *C*_in_ = 45 g L^−1^, *P*_u_ = 280 W, *ω*_s_ = 2250 r.p.m, *t*_ex_ = 4 h). The low-magnification TEM image shown in Fig. 3a indicates that the FL-MoS_2_ nanosheets are individually dispersive and have a lateral size of 161 ± 66 nm according to the statistical data based on the TEM images. The enlarged TEM images in Fig. 3b–d, g show that the MoS_2_ nanosheets are very thin judged by the contrast. Figure 3e shows a selected area electron diffraction (SAED) pattern of a typical MoS_2_ nanosheets of Fig. 3d. The ordered diffraction lattice indicates the MoS_2_ nanosheet is single-crystal and can be readily indexed to a 2H-MoS_2_ phase. Its corresponding high-resolution TEM (HRTEM) image is shown in Fig. 3f, in which the highly ordered crystal lattice corroborates that the FL-MoS_2_ nanosheets, obtained via the CUM process, are of a perfect single-crystal nanostructure [50, 52]. The SAED pattern in Fig. 3h, corresponding to the lower particle of Fig. 3g, consists of two sets of ordered diffraction lattices, suggesting that two single-crystal MoS_2_ nanosheets are assembled together. Figure 3i shows the HRTEM image of the particle corner in Fig. 3g and no clear defects can be found in a large area [68]. From the TEM observations, one can conclude that the MoS_2_ sample obtained by exfoliating natural molybdenite powders via the CUM process takes on a thin plate-like morphology with lateral size of ~160 nm, and the FL-MoS_2_ nanosheets are of a perfect single-crystal structure.

**Fig. 4:**
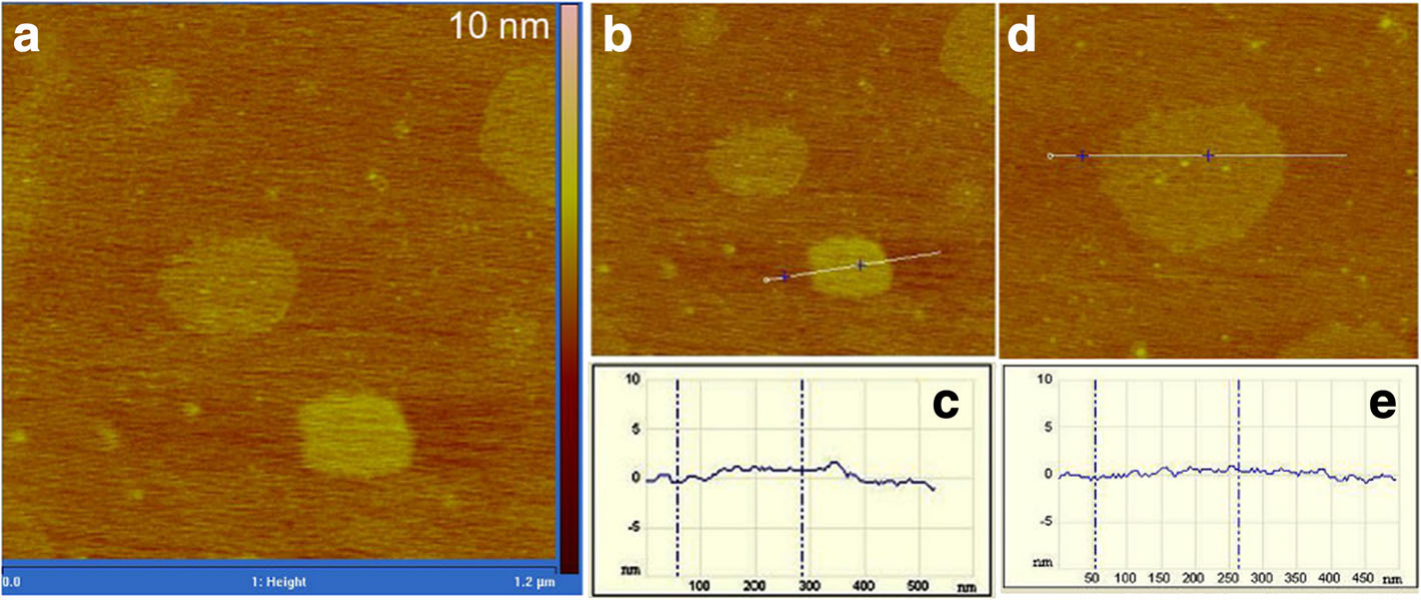
Typical AFM images of the FL-MoS_2_ nanosheets derived from natural molybdenite powders (*R*_pm_ = 1/2, *C*_in_ = 45 g L^−1^, *P*_u_ = 280 W, *ω*_s_ = 2250 r.p.m, *t*_ex_ = 4 h): **a**, **b**, **d** images, **c** a highth scan of the particle in **b**, and **e** a highth scan of the particle in **d**

Figure 4 shows the typical AFM images of the FL-MoS_2_ derived from natural molybdenite powders via the CUM process (*R*_pm_ = 1/2, *C*_in_ = 45 g L^−1^, *P*_u_ = 280 W, *ω*_s_ = 2250 r.p.m, *t*_ex_ = 4 h) with centrifugation at 10,000 r.p.m. The AFM image in Fig. 4a shows the lateral size of the MoS_2_ nanosheets is about 100–250 nm, agreeing with the TEM observations (Fig. 3). Figure 4b–e show the AFM images of some typical MoS_2_ nanosheets with the corresponding height profiles (Fig. 4c, e). According to Fig. 4c, e, the thickness of the MoS_2_ nanosheets is about 1 nm, slightly larger than the thickness (~0.8 nm) of a single MoS_2_ layer exfoliated mechanically [43, 69]. This may be due to the presence of adsorbents on the nanosheets [70]. The AFM results, together with the TEM observations, corroborate that FL-MoS_2_ nanosheets, even single-layer ones, are successfully achieved via the CUM process using natural molybdenite powders as the starting materials.

**Fig. 5:**
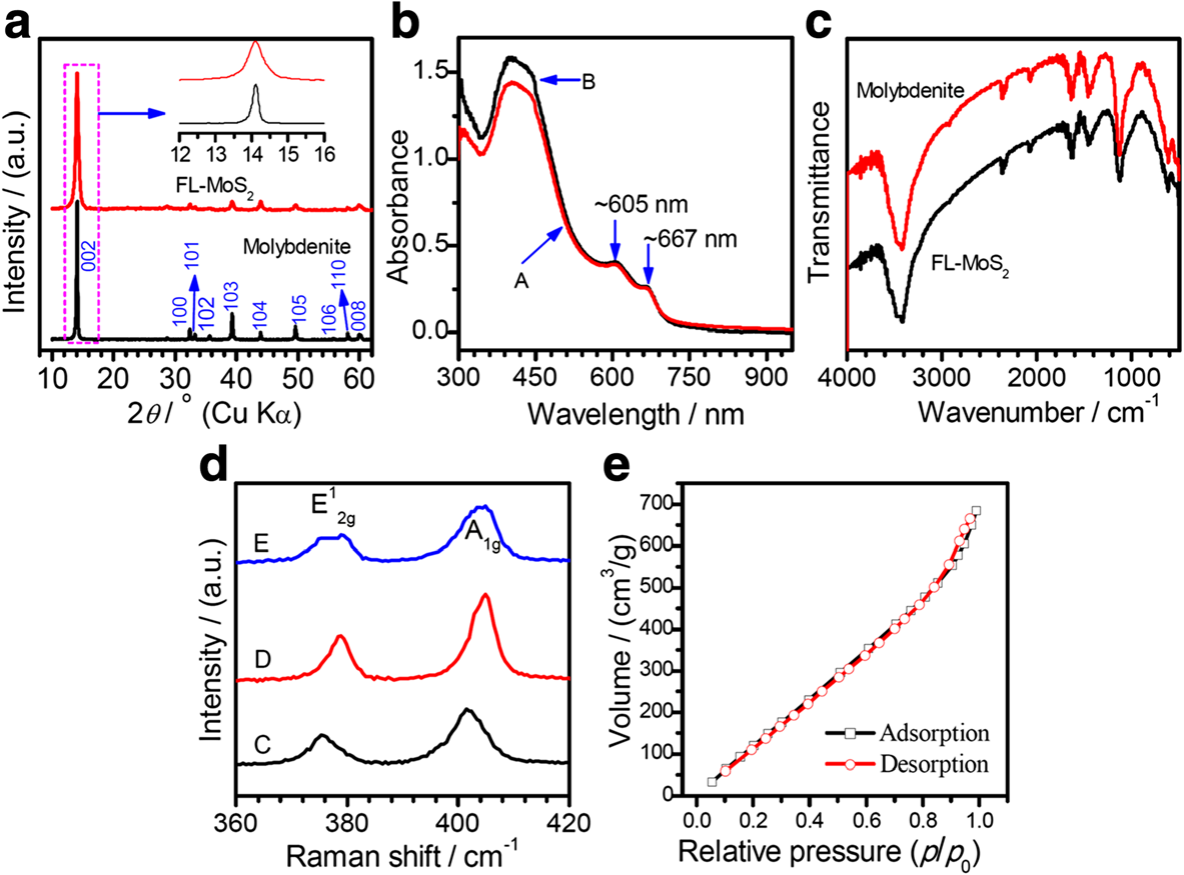
**a** XRD patterns with normalized intensities of the pristine natural molybdenite and the exfoliated FL-MoS_2_ nanosheets obtained by centrifugation between 8000 and 10,000 r.p.m; **b** The UV-vis absorption spectra of (A) the FL-MoS_2_ suspension (the supernatant of the centrifugation at 8000 r.p.m) and (B) the FL-MoS_2_ suspension after standing for 42 days; **c** FT-IR spectra of the FL-MoS_2_ nanosheets obtained by centrifugation at 1500–8000 r.p.m and the pristine natural molybdenite; **d** Raman spectra for the pristine natural molybdenite (C) and the FL-MoS_2_ nanosheets (centrifugation at 1500–8000 r.p.m (D) and 8000–10,000 r.p.m (E), respectively, on a SiO_2_/Si substrate); **e** The N_2_ adsorption-desorption isotherms of the FL-MoS_2_ nanosheets (8000–10,000 r.p.m, *S*_BET_ = 923.57 m^2^ g^−1^). All samples are obtained at the condition of *R*_pm_ = 1/2, *C*_in_ = 45 g L^−1^, *P*_u_ = 280 W, *ω*_s_ = 2250 r.p.m, and *t*_ex_ = 4 h

Figure 5a shows the XRD patterns of the pristine natural molybdenite powders and the FL-MoS_2_ nanosheets exfoliated by the CUM process with centrifugation at 8000–10,000 r.p.m. For the natural molybdenite powders, the diffraction peaks at around 2*θ* = 14.2°, 32.4°, 33.23°, 35.6°, 44.0°, 50.0°, and 58.0° are assignable to the deflections from the planes of (002), (100), (101), (102), (104), (105), and (110) of a 2H-molybdenite (β-MoS_2_ or 2H-MoS_2_) with a hexagonal structure (space group: P6_3_/mmc [194]) according to PDF # 37-1492. The cell parameters can be calculated to be *a* = 0.3181(3) nm, *c* = 1.234(1) nm, and cell vol. = 0.1082(2) nm^3^ using a program UnitCell (a method of TJB Holland and SAT Redfern 1995). The FL-MoS_2_ nanosheets show a similar XRD pattern, suggesting that the phase structure is not changed during the exfoliation by the CUM process, but the (002) peak broadens obviously. The calculated cell parameters of the FL-MoS_2_ nanosheets are *a* = 0.3180(4) nm, *c* = 1.235(2) nm, and cell vol. = 0.1081(3) nm^3^, similar to those of the natural molybdenite powders. The obvious broadening of the (002) diffraction peak after exfoliation indicates the FL-MoS_2_ nanosheets obtained by the CUM process have a small thickness [54], agreeing with the TEM, SEM, and AFM results.

Figure 5b shows the UV-vis spectra of the FL-MoS_2_ dispersions, i.e., (A) the supernatant just obtained by centrifugation at 8000 r.p.m for 45 min and (B) the one after standing for 42 days. One can find that the UV-vis absorption intensities at 605 and 667 nm of the FL-MoS_2_ dispersions before and after standing for 42 days are almost the same. Also, the color of the dark-green FL-MoS_2_ dispersion was not changed during the standing, and no sediment can be found at the bottom of the FL-MoS_2_ dispersion. The peaks at 667 and 605 nm arise from direct excitonic transitions at the K point of the Brillouin zone, and those at ~450 and ~400 nm can be assigned to the direct excitonic transition of the M point [65, 71]. This result confirms that the FL-MoS_2_ dispersion obtained by the CUM process is highly stable. Figure 5c shows the typical FT-IR spectra of the FL-MoS_2_ nanosheets and their precursor (i.e., the pristine molybdenite powder). The band at 475 cm^−1^ correspond to the characteristic vibration of the Mo-S bond due to the coupling vibration of Mo-S group, and the band at 3419 cm^−1^ corresponds to the stretching vibration of water molecules adsorbed on the surface, similar to literature [66]. The FL-MoS_2_ nanosheets and the pristine molybdenite powders have similar bands in their FT-IR spectra, indicating that no impurities were introduced during the exfoliation via the CUM process.

Raman spectra are also used to characterize the thickness of FL-MoS_2_ nanosheets. Figure 5d shows the Raman spectra of the pristine molybdenite powders and the solid FL-MoS_2_ nanosheets obtained after centrifugation at different centrifuging speeds (i.e., one FL-MoS_2_ sample obtained by centrifuging the supernatant of 1500 at 8000 r.p.m and the other one by centrifuging the supernatant of 8000 at 10,000 r.p.m). For the pristine molybdenite powders, a typical peak at 374.4 cm^−1^, known as E^1^_2g_, originates from the in-plane vibration of the Mo-S bond, and an A_1g_ peak at 400.4 cm^−1^ from out-of-plane vibrations is also observed [53]. After exfoliation by the CUM process, the E^1^_2g_ and A_1g_ peaks of the FL-MoS_2_ nanosheets shift towards higher frequency (i.e., blueshift), and the peak difference between E^1^_2g_ and A_1g_ is about 25 cm^−1^. The blueshift of peak A_1g_ can be used to estimate the layer number of MoS_2_ nanosheets [69]. The higher centrifugation speed achieves smaller FL-MoS_2_ nanosheets. The blueshift of Raman shift indicates that the MoS_2_ nanosheets exfoliated by the CUM process consist of a large amount of thin MoS_2_ nanosheets with few layers [9].

Figure 5e shows the N_2_ adsorption-desorption isotherms of the FL-MoS_2_ nanosheets obtained by centrifuging the supernatant of 8000 at 10,000 r.p.m. The FL-MoS_2_ nanosheets do not show a porous microstructure. The BET specific surface area of the FL-MoS_2_ nanosheets is as high as 924 m^2^ g^−1^, whereas their precursor of the pristine molybdenite is only 29 m^2^ g^−1^.

**Fig. 6:**
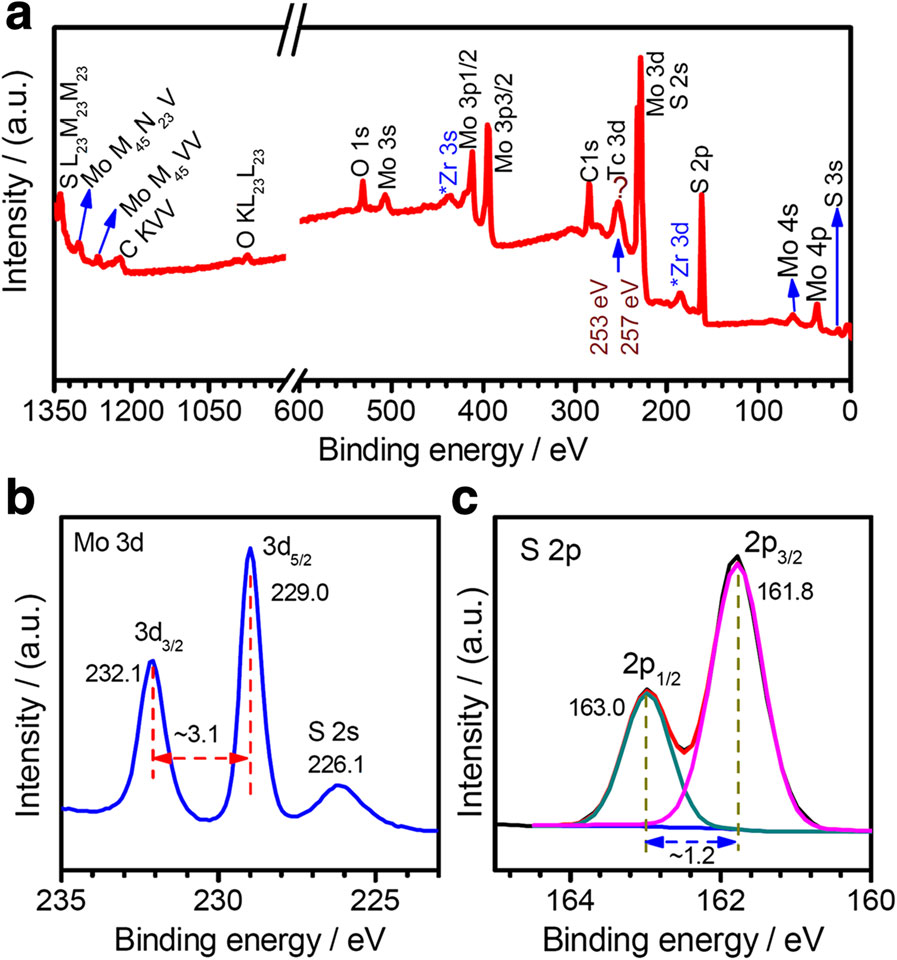
X-ray photoelectron spectroscopy (XPS) of the FL-MoS_2_ nanosheets (1500–8000 r.p.m): **a** a survey scan, **b** Mo 3d, and **c** S 2p

X-ray photoelectron spectroscopy (XPS) was further used to characterize the chemical compositions of the FL-MoS_2_ nanosheets derived by the CUM process. Figure 6 shows the XPS spectra of the FL-MoS_2_ nanosheets obtained by centrifuging the supernatant of 1500 at 8000 r.p.m for 45 min. Figure 6a shows the survey. The major XPS peaks can be assignable to the elements of Mo, S, O, and C, and some weak peaks may correspond to Zr and Tc elements. Mo and S come from the FL-MoS_2_ nanosheets. The elements of O and C are due to the substances (e.g., PVP, CO_2_) adsorbed on the samples. The possible elements of Zr and Tc may come from the ultrasonication horn in the CUM system. Figure 6b shows the core XPS spectrum of Mo 3d. The strong peaks at around 229.0 and 232.1 eV correspond to Mo^4+^ 3d_5/2_ and Mo^4+^ 3d_3/2_, respectively, and the weak peak at 226.1 eV corresponds to S 2s [72]. Figure 6c shows the core XPS spectrum of S 2p, and the peaks at 163.0 and 161.8 eV correspond to the S 2p_1/2_ and S 2p_3/2_ orbitals of divalent sulfide ions, respectively. According to the XPS spectra, Mo and S in the FL-MoS_2_ are in good agreement with the binding energies of Mo^4+^ and S^2−^ ions in the 2H-MoS_2_ phase.

### Factors Influencing the Exfoliation in the CUM Process

**Fig. 7:**
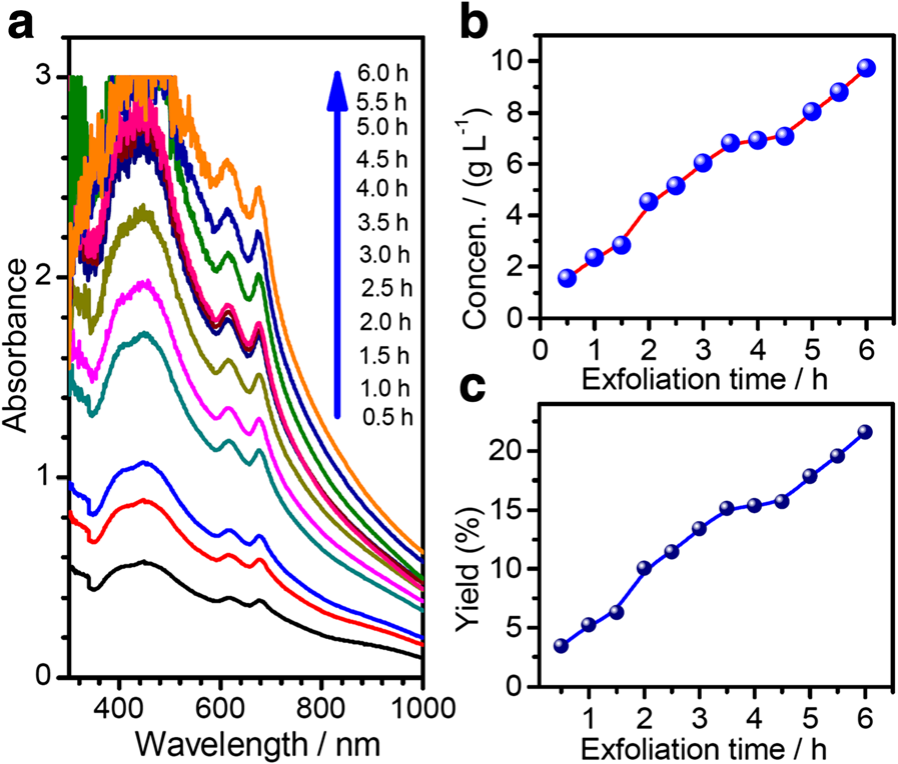
**a** The UV-vis-IR spectra of the FL-MoS_2_ suspensions (*R*_pm_ = 1/2, *C*_in_ = 45 g L^−1^, *P*_u_ = 280 W, *ω*_s_ = 2250 r.p.m) with various exfoliation times (*t*_ex_); **b** the concentrations, and **c** yield of the FL-MoS_2_ suspensions after different exfoliation times

**Fig. 8:**
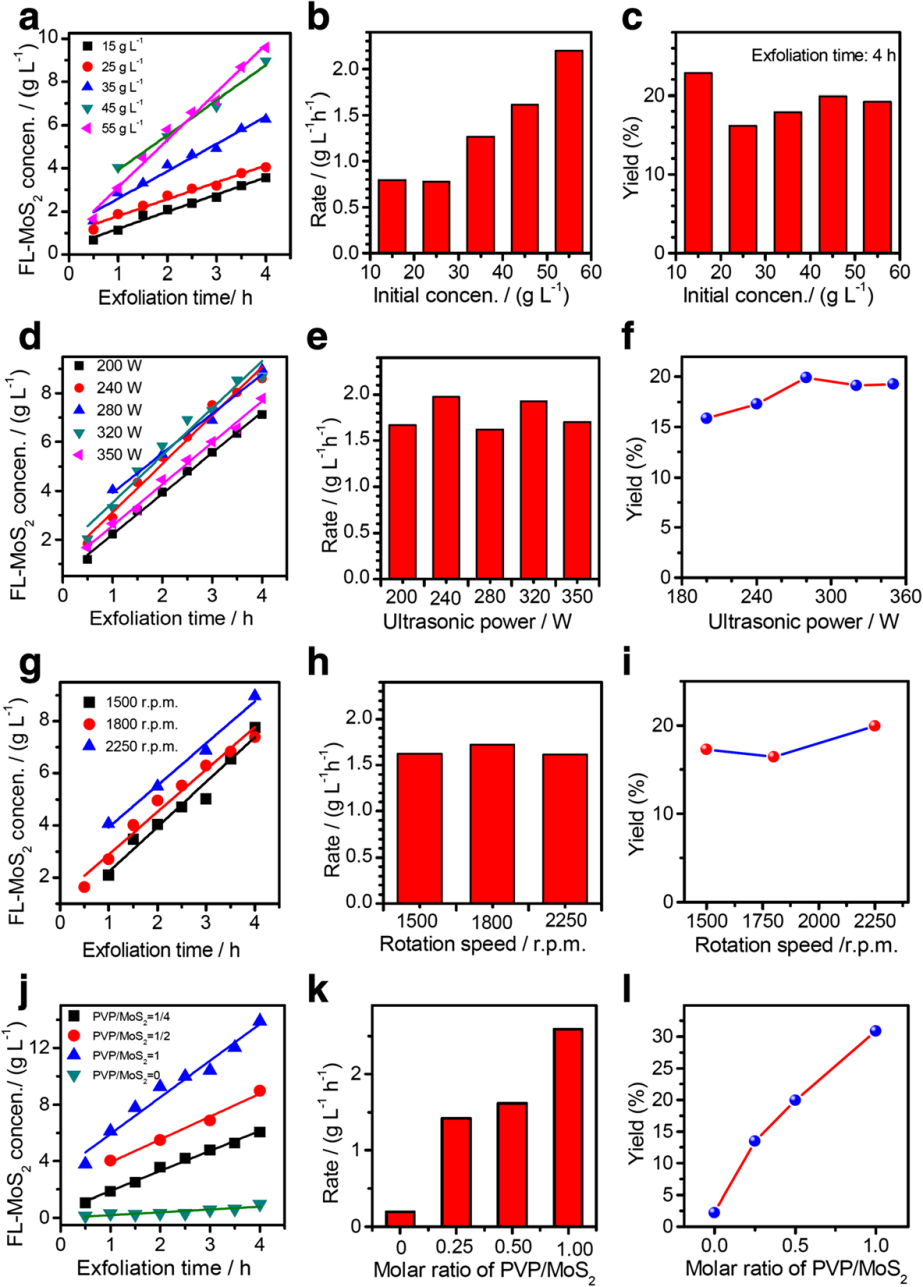
The effects of exfoliating parameters on the supernatant concentration (*C*_ex_), exfoliation rate and yield of FL-MoS_2_ nanosheets from natural molybdenite powders: **a**–**c** the initial concentration of natural molybdenite (*C*_in_ = 15–55 g L^−1^ when *P*_u_ = 280 W, *ω*_s_ = 2250 r.p.m, *R*_pm_ = 1/2); (**d**–**f**) ultrasonic powers (*P*_u_ = 200–350 W when *C*_in_ = 45 g L^−1^, *ω*_s_ = 2250 r.p.m, *R*_pm_ = 1/2); **g**–**i** rotation speeds (*ω*_s_ = 1500–2250 r.p.m when *C*_in_ = 45 g L^−1^, *P*_u_ = 280 W, *R*_pm_ = 1/2); **j**–**l** molar ratios of PVP to MoS_2_ (*R*_pm_ = 0–1 when *C*_in_ = 45 g L^−1^, *P*_u_ = 280 W, *ω*_s_ = 2250 r.p.m)

To further understand the exfoliation of natural molybdenite powders using the CUM process, we carefully designed a series of control experiments to check the factors that influence the exfoliation. The concentrations of the FL-MoS_2_ nanosheets in the NMP suspensions were measured using the UV-vis-IR spectra mentioned in the experimental section. The influencing factors we checked included the exfoliation times, the initial concentrations of natural molybdenite powders, ultrasonic power, rotation speed of sand mill, and PVP concentration. Figures 7 and 8 show the typical results.

Figure 7a shows the UV-vis-IR absorption spectra of the FL-MoS_2_ suspensions in the CUM process (*R*_pm_ = 1/2, *C*_in_ = 45 g L^−1^, *P*_u_ = 280 W, *ω*_s_ = 2250 r.p.m) with various exfoliation times (*t*_ex_ = 0.5–6 h). All the UV-vis-IR spectra take on a similar profile and their absorbance values increases with the increase in the exfoliated time. Figure 7b shows the plot of the concentrations of the FL-MoS_2_ nanosheets versus the exfoliation times, obtained according to the Beer-Lambert law. One can see that the concentration of FL-MoS_2_ nanosheets in the NMP suspension increases linearly with the increase in exfoliation time [73]. The slope of the linear-fitting line can be seen as the exfoliation rate, and the exfoliation rate in the present condition reaches 1.42 g L^−1^ h^−1^. Figure 7c shows the yield of FL-MoS_2_ nanosheets at various exfoliation times. The yield increases linearly with the exfoliation time and reaches 21.6 % when the exfoliation time is 6 h.

The typical results on the factors that influence the exfoliation behavior in the CUM process are summarized in Fig. 8. Figure 8a–c show the effect of the initial concentrations (*C*_in_, 15–55 g L^−1^) of natural molybdenite powders on the exfoliation rate and yield of natural molybdenite powders (*P*_u_ = 280 W, *S*_r_ = 2250 r.p.m, *R*_pm_ = 1/2). Figure 8a shows the change in the concentration of the FL-MoS_2_ (i.e., supernatants obtained by centrifuging at 1500 r.p.m for 45 min after settling for more than 24 h) as the exfoliation time increases with various initial concentrations of natural molybdenite powders. The exfoliation rate and yield with various initial concentrations of natural molybdenite powders are shown in Fig. 8b, c, respectively. The increase in the initial concentration of the molybdenite powders is favorable to enhance the exfoliation rate, but the yield is not in a similar case. Low initial concentration (15 g L^−1^) achieves a higher yield (~23 %) of FL-MoS_2_ nanosheets and makes better dispersion of FL-MoS_2_ nanosheets than the higher ones. Considering the exfoliation amount, we chose an initial molybdenite concentration of 45 g L^−1^ for the following exfoliation experiments. Figure 8d–f show the effect of ultrasonic powers (*P*_u_, 200–350 W) on the exfoliation behavior of natural molybdenite powders (*C*_in_ = 45 g L^−1^, *ω*_s_ = 2250 r.p.m, *R*_pm_ = 1/2). One can see that the higher ultrasonic powers are helpful to improve the exfoliation rate and yield, but this effect is not so obvious. When the ultrasound power is 280 W, the exfoliation yield at 4 h reached highest value of ~20 %. As we know, the ultrasound at a low input power corresponds to the stable cavitation, and the knocking on the right sides of plates has little ability to destroy the covalently bonded S-Mo-S, while the knocking on their profiles can easily overcome van der Waals forces between MoS_2_ nanosheets. When the input power increases, the ultrasound corresponds to inertial cavitation, and the increase in the number of cavitation bubbles leads to a small amount of useful bubbles, weakening the knocking intensity [67]. Therefore, too high or too low sonication power is not beneficial to enhance the exfoliation yield. The ultrasonication power is determined at 280 W in the following experiments. Figure 8g–i show the effect of the rotation speed (*ω*_s_, 1500–2250 r.p.m) of the sand mill on the exfoliation rate and yield at similar conditions (*C*_in_ = 45 g L^−1^, *P*_u_ = 280 W, *R*_pm_ = 1/2). The yield has the highest value when the rotation speed is 2250 r.p.m. A higher rotation speed is more favorable to overcome the van der Waals forces between the MoS_2_ layers and then leads to more defects which accelerate the exfoliation with the synergistic effect of ultrasound. Considering the safety and noise of the sand mill, a suitable rotation speed of 2250 r.p.m is chosen. Figure 8j–l show the effect of PVP concentrations (*R*_pm_, 0–1) on the exfoliation behavior (*C*_in_ = 45 g L^−1^, *P*_u_ = 280 W, *ω*_s_ = 2250 r.p.m). The exfoliation rate and yield increase with the increase of the PVP concentration. PVP molecules in the CUM process act both as the dispersing agent and separating agent to prevent aggregation of the exfoliated MoS_2_ nanosheets. But, if the PVP concentration is too high, the viscosity of the suspension increase rapidly, and the high viscosity leads to a terrible fluidity, resulting in a bad effect on the exfoliation [74]. The PVP content used in the CUM process is kept at *R*_pm_ = 1/2 in the present experiments.

**Fig. 9:**
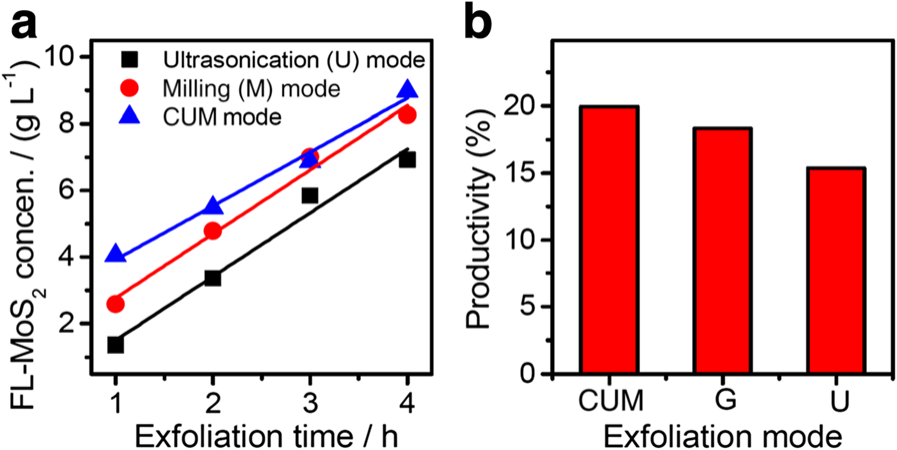
Comparison of the exfoliation yield using different exfoliation mode (*U* ultrasonication, *M* milling, and *CUM* coupled ultrasonication-milling) under the condition of *R*_pm_ = 1/2, *C*_in_ = 45 g L^−1^, *P*_u_ = 280 W and *ω*_s_ = 2250 r.p.m

To demonstrate the synergistic effect of milling and ultrasonication, we comparatively conducted exfoliation experiments under the similar conditions but using different exfoliation modes: coupled ultrasonication-milling (the CUM mode), milling (the M mode) and ultrasonication (the U mode). Figure 9 shows the exfoliation behavior using the three exfoliation modes (*P*_u_ = 280 W, *C*_in_ = 45 g L^−1^, *ω*_s_ = 2250 r.p.m, *R*_pm_ = 1/2). One can see that the CUM process is more efficient in the exfoliation of natural molybdenite powders to form FL-MoS_2_. The exfoliation yield of the CUM process is ~19.9 %, higher than that (18.4 %) of the M process and that (15.4 %) of the U process. The CUM process combines the advantages of both ultrasonic cavitation and shear action in the exfoliation process. During the exfoliation process, the high-speed rotating balls in the M mode knock the edges of the MoS_2_ layers and lead to a large amount of defects and cracks, which are in favor of the insertion of solvent molecules into the interlayer spaces and then overcome the van der Waals forces. Also, the CUM system can realize a continuous process: the milling and ultrasonication can work at the same time. The defects and cracks caused by the milling process are helpful to improve the shearing action of ultrasonication greatly. The shear action and the knocking of cavitation cut down and exfoliate layered molybdenite powders to form smaller and thinner MoS_2_ nanosheets, which are protected by the PVP molecules instantly.

Considering the above results (Figs. 7, 8, and 9), one can safely conclude that the PVP content (*R*_pm_) has the most important effect on the exfoliation of natural molybdenite powders to FL-MoS_2_ nanosheets in the CUM process when compared with the other experimental parameters, including the initial concentration of natural molybdenite powders (*C*_in_), ultrasonic power (*P*_u_), and rotation speed of sand mill (*ω*_s_). The optimal experimental parameters in the CUM process can be determined as *R*_pm_ = 1/2, *C*_in_ = 45 g L^−1^, *P*_u_ = 280 W, and *ω*_s_ = 2250 r.p.m judging from the overall aspects. The exfoliation yield of FL-MoS_2_ nanosheets under the optimal condition for 6 h can reach 21.6 %.

### Electrochemical Performance of FL-MoS_2_ Nanosheets Derived by the CUM Process

**Fig. 10:**
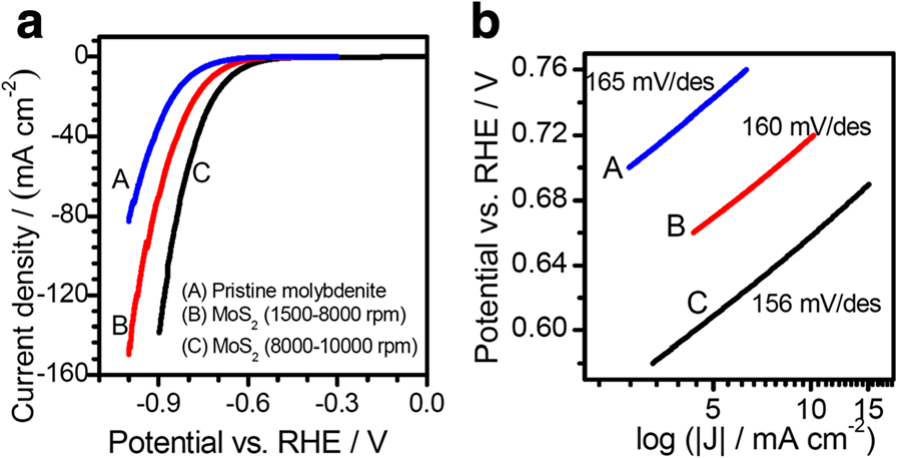
**a** The polarization curves and **b** Tafel plots of the pristine molybdenite (A) and FL-MoS_2_ nanosheets (B: 1500–8000 r.p.m; C: 8000–10,000 r.p.m)

Electrochemical application is one of the most important aspects for the FL-MoS_2_ nanosheets. Figure 10 shows the electrocatalytic performance of the pristine molybdenite powders (A) and the FL-MoS_2_ nanosheets obtained by centrifugation at 1500–8000 r.p.m (B) and at 8000–10,000 r.p.m (C), respectively. Figure 10a shows the typical polarization curves, and their corresponding Tafel plots are shown in Fig. 10b. It is clear that the higher the centrifugation speed, the thinner the FL-MoS_2_ nanosheets are in the same exfoliation using the CUM process. As Fig. 10 shows, the FL-MoS_2_ nanosheets obtained at 8000–10,000 r.p.m (C) have the lowest onset overpotential and the largest exchange current density at a given overpotential among the three samples (A, B, and C). By fitting the Tafel plots linearly according to the equation of *η* = *a* + *b⋅*log|*j*| (where *η* is overpotential, *j* is current density, and *b* is Tafel slope) [75], one can find that the FL-MoS_2_ nanosheets obtained at 8000–10,000 r.p.m have a Tafel slope of 156 mV dec^−1^, lower than that (165 mV dec^−1^) of the pristine molybdenite powders and that (160 mV dec^−1^) of the FL-MoS_2_ nanosheets obtained at 1500–8000 r.p.m. We note that the Tafel slope of the FL-MoS_2_ nanosheets obtained by the CUM process is a little larger than that of some MoS_2_ nanocrystals reported [30, 76]. For examples, a Tafel slope of ~120 mV dec^−1^ was reported for the MoS_2_ nanomaterials synthesized at a temperature of ~500 °C [76] and 140–145 mV dec^−1^ at a temperature of ~850 °C [30]. The better HER performance of MoS_2_-based composites has also been reported, resulting in a Tafel slope of 87 mV dec^−1^ for MoS_2_ and 51 mV dec^−1^ for O-MoS_2_/G [26]. The above samples were synthesized through a conventional hydrothermal reaction using fine chemical reagents as the starting materials. Also, hybridization with other species is another reason to improve their HER performance. Therefore, fine control in the size and morphology (i.e., more edge sites and smaller size) of the MoS_2_ nanosheets and hybridization with other functional species (i.e., graphene) are the possible directions to improve the electrochemical performance of the FL-MoS_2_ nanosheets obtained via the present CUM process from natural molybdenite. The larger Tafel slope obtained in the present work may be due to the difference of the natural molybdenite from other synthetic MoS_2_ in phase compositions. The low electrical conductivity, the possible re-stacking of FL-MoS_2_ nanosheets, the residue of PVP molecules, and the relatively high-temperature treatment in electrode preparation are also possible reasons that lead to a larger Tafel slope. To improve the electrical conductivity, carbon can be used to modify FL-MoS_2_ nanosheets to design highly efficient electrocatalysts for hydrogen evolution reaction and to obtain a lower Tafel slope (47 mV dec^−1^) [77]. Functional modification of the FL-MoS_2_ nanosheets is under way.

**Fig. 11:**
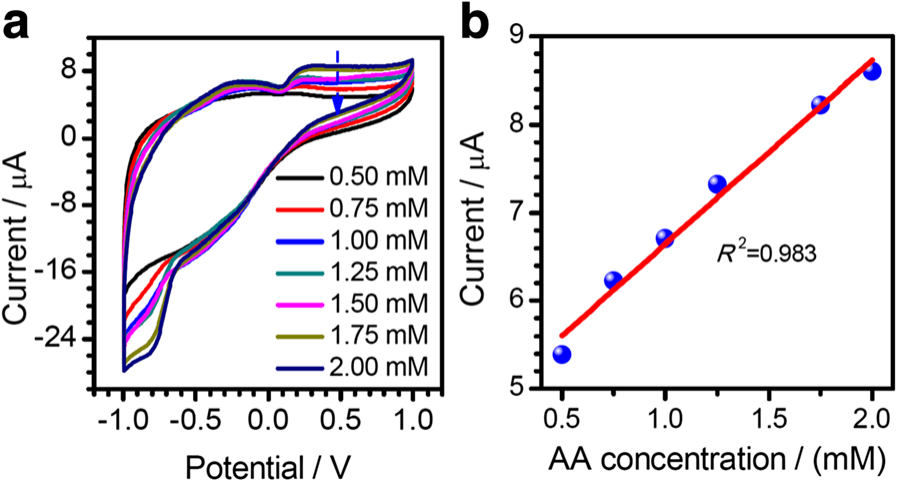
**a** CV curves of the GC electrode modified with the FL-MoS_2_ nanosheets in the presence of ascorbic acid (AA) aqueous solutions with different concentrations ([KCl] = 0.1 M, scan rate = 50 mV s^−1^); **b** The plots of the intensities of the anodic peak current against the ascorbic acid concentration

The electrochemical sensing performance of the FL-MoS_2_ nanosheets was also investigated. Figure 11a shows the CV curves of the GCE modified with the FL-MoS_2_ nanosheets obtained at 8000–10,000 r.p.m in the presence of ascorbic acid (AA) aqueous solutions with different concentrations ([AA] = 0.5–2 mM, [KCl] = 0.1 M, scan rate = 50 mV s^−1^). Figure 11b shows the plots of the intensities of the anodic peak current versus the ascorbic acid concentration. The intensity of the CV peak current increases obviously with the increase in the concentration of ascorbic acid. The linear regression equation of the anodic peak obtained according to the CV curves in Fig. 11a is *I* (uA) = 2.08 (Vs^−1^) · [AA] (mM) – 4.5605, and *R*^2^ = 0.98. The high correlation coefficient suggests a nice linearity correlation between the anodic peak current and ascorbic acid (AA) concentrations in a range of 0.5–2 mM. Unfortunately, the biosensor is of a poor performance, which is possibly caused by the low electrical conductivity of the MoS_2_ nanosheets. To anchor a second-phase particles on the MoS_2_ nanosheets is under our consideration to enhance their electrochemical sensing properties. Actually, MoS_2_ nanosheets have been used as the supporting material to stabilize metal nanoparticles (e.g., Au) to form hierarchical nanocomposites for the detection of chemical and biological molecules [78]. The FL-MoS_2_ nanosheets exfoliated by the CUM process can be a promising active material or support to construct electrochemical electrodes for the electrochemical detection of chemical and biological molecules.

## Conclusions

In summary, a novel coupled ultrasonication-milling (CUM) process has been developed for the first time to exfoliate the natural molybdenite powders to synthesize FL-MoS_2_ nanosheets in the NMP solution using PVP as the exfoliation agent. The factors that influence the exfoliation behavior during the CUM process have been systematically investigated. The FL-MoS_2_ nanosheets obtained at the optimal conditions (i.e., initial molybdenite concentration = 45 g L^−1^, ultrasonic power = 280 W, rotation speed of sand mill = 2250 r.p.m, and the mole ratio of PVP to MoS_2_ = 0.5), taken on a thin single-crystal plate-like morphology, several nanometers in thickness and hundred nanometers in width. The as-obtained FL-MoS_2_ nanosheets have a high specific surface area of 924 m^2^ g^−1^, much larger than that (29 m^2^ g^−1^) of their precursor (i.e., molybdenite powders). The yield of FL-MoS_2_ nanosheets obtained by the CUM process for 6 h reaches to 21.6 %, much larger than that of the literature reported. Also, the CUM process has a higher yield than the ultrasonication (U) or milling (M) process, confirming that the synergistic effect of the ultrasonication and milling process is obvious in the exfoliation of natural molybdenite powders. The as-obtained FL-MoS_2_ nanosheets show highly enhanced electrocatalytic performance in hydrogen evolution reaction and good electrochemical sensing property in detecting ascorbic acid when compared with their precursor. The exfoliation of natural molybdenite via the coupled ultrasonication-milling process developed here provides a novel, green, efficient, scalable, and cost-effective approach to prepare FL-MoS_2_ nanosheets that are of promising applications as electrocatalytic and sensing-active materials.

## Abbreviations

CUM: Coupled ultrasonication-milling
FL-MoS_2_: Few-layer MoS_2_
LPE: Liquid-phase exfoliation
M: Milling
MoS_2_: Molybdenum disulfide
NMP: *N*-methyl-2-pyrrolidone
PVP: Polyvinylpyrrolidone
U: Ultrasonication
